# The role of electrocochleography and the caloric test in predicting short-term recurrence of benign paroxysmal positional vertigo

**DOI:** 10.3389/fneur.2023.1225857

**Published:** 2023-08-23

**Authors:** Yanmei Zhang, Yueqi Wang, Zhen Zhen, Junbo Zhang, Zhengang Zeng, Zhen Zhong, Quangui Wang

**Affiliations:** Department of Otolaryngology, Head and Neck Surgery, Peking University First Hospital, Beijing, China

**Keywords:** endolymphatic hydrops, canal paresis, benign paroxysmal positional vertigo, recurrence, inner ear function

## Abstract

**Objective:**

The study aimed to assess the value of physiological tests for evaluating inner ear function in predicting the short-term recurrence of benign paroxysmal positional vertigo (BPPV).

**Materials and methods:**

The clinical information of all idiopathic BPPV patients who were treated in our clinic between February 2021 and December 2022 were reviewed. All patients included in the study had completed audiology examinations including pure tone audiometry, electrocochleography (EcochG), auditory brainstem response, and vestibular function examination such as the vestibular caloric test. The relationships between the results of the above tests and short-term recurrent BPPV were analyzed.

**Results:**

A total of 96 patients with unilateral idiopathic BPPV were included for analysis. The numbers of non-recurrent patients and recurrent patients were 57 (59.4%) and 39 (40.6%), respectively. Only the results of EcochG and the caloric test showed significant differences between non-recurrent and recurrent patients (both *P* < 0.05). The results of these two tests were also found to be independently predictive of short-term recurrence (both *P* < 0.05). The non-recurrence rate for patients with normal results in both tests reached up to 78.3%, which was significantly higher than that for patients with abnormal results in both tests, 28.6% (*P* < 0.05).

**Conclusion:**

Endolymphatic hydrops and canal paresis were independent risk factors for short-term recurrent BPPV. Additional treatments should be considered to reduce the recurrence rate, including dehydration treatment and vestibular rehabilitation.

## Introduction

Benign paroxysmal positional vertigo (BPPV) is a common labyrinthine disorder caused by mechanical stimulation of the vestibular receptors within the semicircular canals. It is a peripheral vestibular end-organ disease elicited by specific head positions or changes in head position relative to the direction of gravity, characterized by recurrent transient vertigo and typical nystagmus. BPPV is certainly the most common cause of vertigo in adults, and its lifetime cumulative incidence in the general population amounts to ~10% (1). In most cases, BPPV is idiopathic but can also be secondary to head trauma and various disorders of the inner ear. BPPV may impair patients' daily activities and increase their risk of falling.

BPPV is prone to relapse. The recurrence of BPPV has been defined as the reappearance of positional vertigo and nystagmus after at least 1 month from the execution of an effective canalith repositioning treatment (CRT) (2). The recurrence rate of this disease has been reported to range from 13.7 to 65% (2–4). However, it is difficult to predict and may cause stress and anxiety to patients.

Several systemic factors are believed to be correlated with BPPV recurrence, including osteoporosis, vitamin D deficiency, diabetes mellitus, hypertension, hyperlipidemia, cardiovascular disease, and thyroid disorders (2, 5, 6). These studies generally focused on the effect of patients' general physical health on prognosis. However, few researchers have explored the relationship between the local conditions of the inner ear and BPPV prognosis.

BPPV is a labyrinthine disease of the inner ear. Theoretically speaking, the impairment of inner ear function should have a closer relationship with BPPV prognosis. The recurrence of BPPV, especially the short-term recurrence, may be attributed to some kind of inner ear function impairment. Electrocochleography (EcochG), auditory brainstem response (ABR), and the caloric test are the most commonly used physiological techniques for evaluating the function of the inner ear in our clinic. Accordingly, the present study aimed to assess the value of these tests in predicting the short-term recurrence of BPPV, which may provide some references for clinical treatments.

## Materials and methods

### Study population

This is a retrospective study. All clinical data are from our hospital, a tertiary hospital affiliated with a university. From February 2021 to December 2022, 423 adult patients (≥18 years) were diagnosed with BPPV in our outpatient clinic according to the BPPV diagnostic criteria published by the Committee for the Classification of Vestibular Disorders of the Bárány Society in 2015. At the first visit, clinical data were collected, which included age, gender, location of BPPV, chronic disease (osteoporosis, diabetes, hypertension, hyperlipidemia, thyroid disorders, etc.), and previous history of vertigo. In this study, patients with neurological disorders and various inner ear disorders were excluded, including Meniere's disease, sudden deafness, ear surgery, and inner ear trauma. Only patients with idiopathic BPPV were included.

The locations of BPPV were determined by the physical examination with a positive result on the Dix–Hallpike maneuver or roll test. All BPPV patients were treated with CRT according to the locations of BPPV (7–9). Patients were considered cured if they became completely free of signs and symptoms after a CRT. All cured patients were advised to return to the clinic 1 month after treatment. Patients were also instructed to return to the clinic immediately if they suspected BPPV relapse. In this study, the short-term recurrence of BPPV was defined as the reappearance of positional vertigo and nystagmus after at least 1 month but within 3 months from the initial effective CRT. During the follow-up period, physiological tests in otology were performed on the patients, including pure tone audiometry (PTA), tympanometry, EcochG, ABR, and the caloric test.

### Audiometric evaluation

PTA was recorded using a clinical audiometer (Interacoustics AC 40, Denmark) in a sound insulation room. Pure tone thresholds were measured in each ear separately at frequencies of 250–8000 Hz for air conduction with TDH-39 headphones and 250–4000 Hz for bone conduction with a “Radio Air B-71” bone transducer in all participants. Pure tone averages were calculated by determining the mean value of hearing thresholds at 500, 1000, 2000, and 4000 Hz. Hearing loss was defined as an average hearing threshold of > 25 dBHL. Tympanometry was performed by using a clinical impedance audiometer (Interacoustics AT 235, Denmark) with a 226 Hz probe tone. Patients with type A tympanogram were included in the study.

### EcochG and ABR

Both EcochG and ABR were recorded using an auditory-evoked potential device (ICS CHARTR, GN Otometrics) and ER3A inserted headphones in an acoustic electromagnetic shielding room. The stimuli for EcochG consisted of 100 ms alternating clicks, summing to 500 sweeps, presented monaurally at rates of 11.1/s with 95 dB nHL intensity levels. EcochG was performed using tympanic recording and was considered positive if the negative summating potential/active potential (SP/AP) amplitude ratio was ≥0.36 in our hospital.

The stimuli for ABR consisted of 100 ms alternating clicks, summing to 2000 sweeps, presented monaurally at rates of 11.1/s and 55.1/s with 80 dB nHL intensity levels. Absolute latency on waves I, III, and V and interpeak latency on waves I–III, III–V, and I–V were recorded at low and high rates. Then, for each value, the values obtained at the low and high rates were subtracted from each other. A difference in interpeak latency waves I–V beyond 0.28 ms was considered abnormal (10).

### Caloric test

The caloric test was performed using video nystagmography (VNG) in a dark room. Ice water (20°C; 20 mL) was injected into the external auditory meatus over 20 s in turns, and the induced nystagmus was recorded in a dark, open-eyes situation. When the maximum slow phase eye velocity of bilateral eyes was < 6°/s, the ice water (0°C) irrigation test was added. Canal paresis (CP) was judged as positive when the maximum slow phase eye velocity was ≤ 6°/s or the bilateral asymmetry ratio was ≥25%.

### Statistical analysis

The statistical analysis in the current study was performed with SPSS software version 22.0 (IBM, Armonk, NY). Continuous variables were all displayed as mean ± standard deviation. The unpaired Student *t*-test and one-way ANOVA were used to compare continuous variables between different groups. The multiple regression analysis was used to identify significant associations. For all analyses, a *P*-value of < 0.05 was considered to be statistically significant.

## Results

Among 423 adult BPPV patients, 226 patients returned to the outpatient clinic for follow-up. Only 104 patients completed all electrophysiological tests. A total of eight patients with bilateral BPPV were excluded as they represented an excessively small sample size. Therefore, a total of 96 patients with unilateral idiopathic BPPV were finally included for analysis. The numbers of non-recurrent and recurrent patients were 57 (59.4%) and 39 (40.6%), respectively. The baseline information according to treatment outcomes is shown in Table 1: only the results of EcochG and the caloric test showed significant differences between non-recurrent and recurrent patients (both *P* < 0.05).

**Table 1:** Baseline information of the 96 patients according to short-term treatment outcomes.

	**All patients (*n =* 96)**	**Non-recurrent patients (*n =* 57)**	**Recurrent patients (*n =* 39)**	***P*-value**
Sex				0.530
Male	28	18	10	
Female	68	39	29	
Age (years)	57.6 ± 13.1	57.1 ± 12.7	58.3 ± 13.7	0.665
Chronic disease				0.099
Yes	13	5	8	
No	83	52	31	
Affected side				0.338
Left	35	23	12	
Right	61	34	27	
Affected semicircular canal				0.789
Posterior	75	44	31	
Horizontal	21	13	8	
PTA of affected ears (dB)	22.1 ± 13.1	20.4 ± 12.1	24.5 ± 14.4	0.141
PTA of contra-lateral ears (dB)	22.9 ± 15.6	21.7 ± 14.9	24.6 ± 16.6	0.371
EcochG				0.011^*^
Normal	59	41	18	
Abnormal	39	16	21	
Caloric test				0.001^*^
Normal	69	48	21	
Abnormal	27	9	18	
High-rate ABR				0.775
Normal	68	41	27	
Abnormal	28	16	12	

* Statistically significant.

All the variables listed in Table 1 were also analyzed using multiple regression analysis to identify the factors that could determine treatment outcomes. The results also suggested that only the results of EcochG (*P* = 0.025) and the caloric test (*P* = 0.001) could be included. The OR value was 2.899 for EcochG, with a 95% confidence interval (CI) of 1.144–7.346, and the OR value was 6.473 for the caloric test, with a 95% CI of 2.053–20.408.

The distribution of treatment outcomes according to the combination of these two tests is given in Table 2. There was a significant difference between these groups of patients with respect to non-recurrence rates (*P* = 0.002). The non-recurrence rate for patients with normal results in both tests reached up to 78.3%, while it was only 28.6% for patients with abnormal results in both tests.

**Table 2:** Distribution of treatment outcomes according to the combination of EcochG and caloric test.

	**N. of patients (non-recurrent/recurrent)**	**Non-recurrence rate (%)**	***P-*value**
Group^#^			0.002^*^
Group 1	46 (36/10)	78.3	
Group 2	23 (12/11)	52.2	
Group 3	13 (5/8)	38.5	
Group 4	14 (4/10)	28.6	

# Group 1: patients with normal results of both EcochG and caloric test; Group 2: patients with abnormal results of EcochG and normal results of caloric test; Group 3: patients with normal results of EcochG and abnormal results of caloric test; Group 4: patients with abnormal results of both EcochG and caloric test.

* Statistically significant.

## Discussion

In this study, the clinical data and inner ear physiological data that might affect the short-term recurrence of BPPV were analyzed. The results of multiple regression analysis showed that EcochG test outcomes and caloric test outcomes were independent risk factors for recurrence, while factors such as gender, age, chronic disease, affected side, hearing level, high-rate stimulated ABR, and location of BPPV were not. Patients with abnormal SP/AP of EcochG or abnormal CP value of caloric test had a higher recurrence rate than those with normal test outcomes. This indicates that latent inner ear impairment does exist in patients with recurrent BPPV.

In this study, the abnormal EcochG refers to an increased SP/AP ratio. The increased SP/AP ratio is generally associated with endolymphatic hydrops or auditory neuropathy (11–13). In this study, the hearing conditions of the two groups of patients were similar. Therefore, the increased SP/AP ratio indicated that patients with recurrent BPPV were more likely to have endolymphatic hydrops. It was hypothesized that detached saccular otoconia might be a causative factor for hydrops. In the case of BPPV, the endolymphatic compartment appears to contain free-floating debris, comprising protein and mineral particles of varying size, weight, and consistency. Endolymphatic hydrops might be due to the increased water-binding capacity of proteinaceous debris. It would seem that, at least in some cases of endolymphatic hydrops, increased osmotic pressure within the membranous labyrinth may play a role.

On the other hand, the presence of endolymphatic hydrops may also make BPPV intractable (14). Patients with Meniere's disease (i.e., endolymphatic hydrops) often develop BPPV. This could be attributed to the repeated distention of the membranous labyrinth due to hydrops, which may lead to otoconia detachment, loss of resilience, and partial collapse of the semicircular canal. The resulting partial obstruction prevents the otoliths—calcified protein—from returning to the vestibule completely during the repositioning maneuvers, increasing the rate of recurrence. Partial obstruction may also be due to a dilated saccule or the adhesion of otoliths to the membranous labyrinth, leading to an aggravation of the condition (2, 15). Otoconia detachment and endolymphatic hydrops are mutually reinforcing. Endolymphatic hydrops is a potential cause of BPPV occurrence and recurrence. Therefore, it should not be ignored in dealing with BPPV. Once the electrophysiological examination confirms the existence of endolymphatic hydrops, the corresponding treatment should be considered.

The caloric test is a classic technique for evaluating vestibular function and can help identify potential lesions in vestibular organs. Abnormal CP in the caloric test was another independent risk factor for BPPV recurrence. Bi et al. (16) also found that the recurrence rate of BPPV patients with abnormal caloric test results was higher than that of patients with normal caloric test results. Abnormalities in caloric test results are rather frequent in patients with BPPV and require repeated treatment (17–19). The caloric test mostly assesses the function of the horizontal semicircular canal. However, cases of BPPV with posterior canal involvement also displayed abnormal caloric test findings. Korres et al. (17) suggested that some extensive damage to the vestibular end-organ might exist, with more than one canal involved. Another explanation could be that otoconia in the endolymph of the involved canal might cause a gravitational load in the fluid and thus affects the caloric response.

In this study, the recurrence rate was 71.4% if patients had abnormal results in both EcochG and caloric tests (Table 2). When otoconia in the endolymph caused damage to both cochlear and semicircular canals, BPPV was prone to relapse. A high recurrence rate of BPPV is related to more serious damage in the inner ear.

Ischemia of the inner ear may be one of the causes of BPPV (2, 20, 21). The high-rate ABR is more sensitive than typical ABR due to the time characteristics of sound stimulation, making it superior in the diagnosis of ischemia (10). In this study, the high-rate ABR was not an independent risk factor for short-term recurrent BPPV. Compared to factors such as endolymphatic hydrops and impaired semicircular canal function, ischemia of the inner ear may not be the main cause of short-term recurrence. However, Neri et al. (22) demonstrated that ischemia could lead to the development of recurrent BPPV. Therefore, there is another possibility that the high-rate ABR cannot sensitively reflect the presence of inner ear ischemia. To confirm the accuracy and sensitivity of the high-rate ABR in diagnosing inner ear ischemia, rigorous clinical trials are required.

Previous studies have shown that systemic chronic diseases such as diabetes are risk factors leading to the recurrence of BPPV (2, 6). However, the correlation between chronic diseases and the recurrence of BPPV was not reflected in this study. One possibility is that its sample size is relatively small. Another possibility is that systemic diseases such as diabetes affect the prognosis of BPPV treatment outcomes by impairing the function of the inner ear. D'Silva et al. (23) demonstrated that diabetes could cause some damage to the inner ear. Therefore, indicators for evaluating the function of the inner ear may be more sensitive in predicting the short-term recurrence of BPPV than indicators for systemic diseases.

### Limitations

Some limitations must be addressed. First, this is a single-center retrospective study, and the patient sample was relatively small. Second, the video head impulse test (vHIT) is considered a very effective test to evaluate the function of semicircular canals, and patients failed to undergo this examination because our department did not have the equipment at that time. At last, the purpose of this study was to analyze the risk factors influencing the short-term recurrence of BPPV, so the follow-up time was 3 months, which is relatively short.

## Conclusion

Endolymphatic hydrops and CP were independent risk factors for short-term recurrent BPPV. Additional treatments should be considered to reduce the recurrence rate, including dehydration drugs such as hydrochlorothiazide and vestibular rehabilitation training.

## Data availability statement

The raw data supporting the conclusions of this article will be made available by the authors, without undue reservation.

## Ethics statement

The studies involving humans were approved by the Ethics Committee of Peking University First Hospital. The studies were conducted in accordance with the local legislation and institutional requirements. The ethics committee/institutional review board waived the requirement of written informed consent for participation from the participants or the participants' legal guardians/next of kin because this is a retrospective study.

## Author contributions

ZZho and QW designed the study and revised the manuscript. YZ, YW, ZZhe, JZ, and ZZe participated in the material preparation, data collection, data analysis, and drafting of the manuscript. All authors contributed to the article and approved the submitted version.
